# Innate Immune Response of Alveolar Macrophage to House Dust Mite Allergen Is Mediated through TLR2/-4 Co-Activation

**DOI:** 10.1371/journal.pone.0075983

**Published:** 2013-10-01

**Authors:** Chia-Fang Liu, Daniel Drocourt, Germain Puzo, Jiu-Yao Wang, Michel Riviere

**Affiliations:** 1 Centre National de la Recherche Scientifique, Institut de Pharmacologie et de Biologie Structurale, Toulouse, France; 2 Université de Toulouse, Université Paul Sabatier, Institut de Pharmacologie et de Biologie Structurale, Toulouse, France; 3 Institutes of Basic Medicine, National Cheng-Kung University, Tainan, Taiwan; 4 CAYLA-Invivogen, Toulouse, France; 5 Pediatrics, College of Medicine, National Cheng-Kung University, Tainan, Taiwan; University Medical Center Freiburg, Germany

## Abstract

House dust mite, *Dermatophagoides pteronyssinus* (Der p), is one of the major allergens responsible for allergic asthma. However, the putative receptors involved in the signalization of Der p to the innate immune cells are still poorly defined as well as the impact of their activation on the outcome of the allergen-induced cell response. We previously reported that the HDM activation of mouse alveolar macrophages (AM) involves the TLR4/CD14 cell surface receptor complex. Here using a TLR ligand screening essay, we demonstrate that HDM protein extract engages the TLR2, in addition to the TLR4, in engineered TLR-transfected HEK cells but also in the MH-S mouse alveolar macrophage cell line model. Moreover we found that the concomitant recruitment of the MH-S cell’s TLR2 and TLR4 receptors by the HDM extract activates the MyD88-dependent signaling pathway and leads to the secretion of the NF-κB regulated pro-inflammatory factors NO and TNF-α. However unlike with the canonical TLR4 ligand (i.e. the bacterial LPS) mobilization of TLR4 by the HDM extract induces a reduced production of the IL-12 pro-inflammatory cytokine and fails to trigger the expression of the T-bet transcription factor. Finally we demonstrated that HDM extract down-regulates LPS induced IL-12 and T-bet expression through a TLR2 dependent mechanism. Therefore, we propose that the simultaneous engagement of the TLR2 and TLR4 receptors by the HDM extract results in a cross regulated original activation pattern of the AM which may contribute to the Th2 polarization of the allergen-induced immune response. The deciphering of these cross-regulation networks is of prime importance to open the way for original therapeutic strategies taking advantage of these receptors and their associated signaling pathways to treat allergic asthma.

## Introduction

The house dust mite (HDM) *Dermatophagoides pteronyssinus* is among the most common domestic sources of indoor allergens and the cause of about 50% of the allergic asthma cases [1,2].

Although inhalation of HDM is harmless in the vast majority of people, it triggers wheezing, dyspnea, airflow obstruction associated to acute airway hyper-responsiveness (AHR) in sensitized (allergic) patients. These symptoms are attributed to a deviant pathological activation of CD4^+^Th2 T lymphocytes which produce IL-4, IL-5 and IL-13 contributing to the local recruitment of inflammatory cells, up-regulation of the IgE secretion, AHR and airways remodeling [3]. The dysregulation of the airways adaptive immunity induced by the allergen is considered to be largely orchestrated by the pulmonary dendritic cells (DCs) [4]. However the primary detection of the inhaled HDM is ensured by airway tissue structural cells [5] and alveolar macrophages (AMs) which account for up to 90% of the lung hematopoietic cells [6]. These first defense line cells play a determinant, but not yet fully appreciated role in the control or the initiation and propagation of allergic asthma by sensing and transmitting the allergen signal to DCs, but also by contributing to the polarization of the DC dependent and independent lung inflammation [5,7-9]. In a previous work [10], some of us reported that stimulation by HDM extract of two distinct mouse alveolar macrophages cell lines CD14^high^/TLR4^high^ (MH-S) and CD14^low^/TLR4^low^ (AMJ2-C11) induced different level of tumor necrosis factor-α (TNF-α) and nitric oxide (NO) production suggesting a TLR dependent macrophage sensing of HDM extract. However the molecular bases of the TLR dependent macrophage activation by HDM are still poorly defined despite the growing number of indirect but compelling evidences of the crucial impact of the TLR signaling in the physiopathology of allergic asthma [11,12]. Indeed Trompette et al [13] have demonstrated that Der p 2, a major HDM allergen, mimics MD-2 function and directly interacts with TLR4 facilitating the signaling of sub-stoichiometric concentration of naturally inseparable LPS. On the other side, activation of airway smooth muscle cells by Der p 2 has been found to be independent of TLR4 but through a TLR2/MyD88 dependent mechanism [14]. Recently HDM extract has been shown to trigger, as do the TLR ligands zymosan (TLR2-6), LPS (TLR4), and CpG (TLR9), the production by myeloid DCs of the type 1 cytokine TSLP (thymic stromal lymphopoïetin) involved in lymphopoïesis and development of asthma [15]. Finally it has been proposed that HDM extract can distinctly trigger allergic rhinitis through the β-glucan TLR2 dependent activation of the upper airways or allergic asthma through the LPS-induced TLR4 activation of the lower airways [16]. Then face to these independent but converging observations we aimed at identifying definitely which of the TLR pathways are triggered by a whole HDM extract and to analyze at a unique cell population level, the potential contribution of the HDM stimulated macrophage TLR-dependent response to the allergic asthma symptomatology using the previously described mouse alveolar macrophage cell line MH-S model.

## Results

### HDM extract contains bothTLR2 andTLR4 agonists

**Figure 1:**
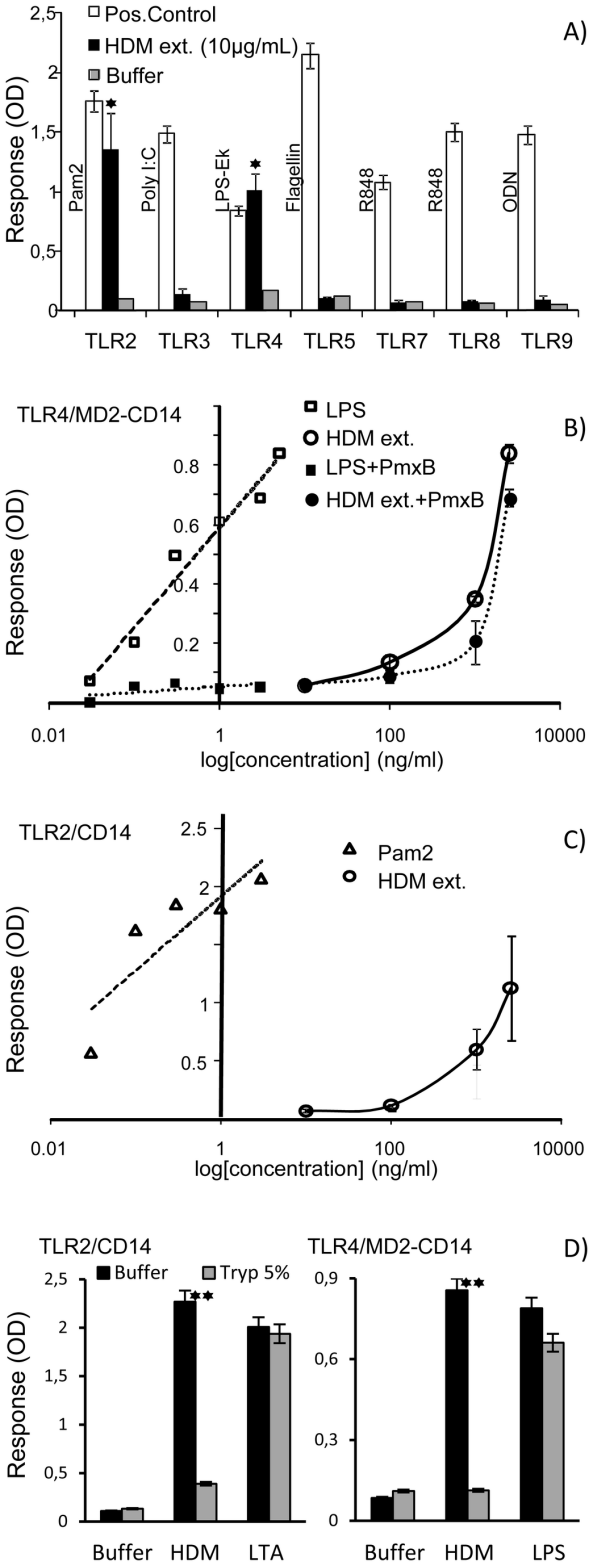
Identification of the TLR pathways involved in the signaling of the HDM extract. (A) responses of the TLR transfected HEK-Blue^TM^ cell lines (Invivogen) stimulated with their respective canonical agonists (positive control: open bars), the HDM extract (black bars) and the medium alone (negative control: grey bars): Pam2: di-palmitoyl-C-S-K_4_ lipopeptide agonist of the TLR1-2complex; Poly I:C : Polyinosinic: polycytidylic acid agonist of TLR3; LPS-Ek: *E. coli* K12 lipopolysaccharide agonist of TLR4, Flagellin ; *B. subtilis* flagellin agonist of TLR5; R848: imidazoquinoline agonist of TLR7 and TLR8; ODN: Unmethylathed CpG oligonucleotide agonist of TLR9. (**B**) Dose response curves of the TLR4/MD2-CD14 co-transfected HEK-Blue^TM^ cell line challenged with the HDM extract (HDM ext.) and the LPS-Ek agonist with (black symbols) or without (open symbols) polymyxin B pretreatment (30min, 10µg/mL). (C) Dose response curves of the TLR2/CD14 co-transfected HEK-Blue^TM^ cell line challenged with the HDM extract (HDM ext.) and the Pam2 agonist. (D) Effect of 5% trypsin treatment (w/w) of the HDM extract and TLR2 and TLR4 agonists (LTA 1µg/mL or *E. coli* LPS, 0.1µg/mL) on their ability to activate the TLR2/CD14 and TLR4/MD2-CD14 co-transfected HEK-Blue^TM^ cell line response. (data are expressed as the means ± SEM of duplicate measures of two independent experiment and the statistical differences were analyzed using Student’s *t*-tests; *: p<0.05; **p<0.01).

To confirm the engagement by HDM of a TLR signalization and to precisely identify the TLR pathways activated, TLR transfected human embryonic kidney reporter cells (HEK-293), engineered to sense individually TLR2, -3, -4, -5, -7, -8, -9 ligands, were challenged with a HDM extract. Owing to this, we found that only the TLR2 or TLR4 transfected cells responded positively to HDM extract (Figure 1A). This result definitively confirms that HDM extract contains factors including certainly but probably not exclusively the reported β-glucan or/and Der p 2 allergen, able to trigger the TLR2 (as TLR2-TLR1 or TLR2-TLR6 heterodimers) and the TLR4 signalling pathways [13,16]. On the other side, it is noteworthy that this observation tends to demonstrate the absence of other TLR ligands and excludes the direct contribution of any other allergen triggered TLR signaling to the allergic response to HDM.

TLR4 signalling activation was further analyzed using TLR4/MD2-CD14 transfected HEK-cells which strongly responded to HDM extract activation (0.9 OD units / 2.5 µgmL^-1^ of HDM extract versus 0.17 OD units with sham buffer) with a level of stimulation comparable to that obtained with approximately 3 orders of magnitude lower concentration of LPS (0.84 OD units / 5 10^-3^ µgmL^-1^) (Fig. 1B). The estimation of the LPS content of the HDM extract via the LAL test, indicates an endotoxin activity of about 1 Endotoxin Unit/mg of HDM extract which correspond to a mass concentration ratio of LPS to HDM of about 10^-4^ (0.25 ng equivalent *E. Coli* O.55:B5 LPS per 2.5 µg of HDM extract according USP EC-6 endotoxin reference standard). Obviously, LPS is active even at so low concentration (0.25 ng/mL) as shown by the dose response curve (Figure 1B). Then to verify whether activation of the HEK-TLR4/MD2-CD14 cells by the HDM extract is due to contaminating LPS or to HDM genuine components, free LPS was depleted by trapping with polymyxin B. Interestingly, while this treatment almost completely abrogates the LPS activity it only affects very slightly the response of the HEK-TLR4/MD2-CD14 cells to the HDM extract (Figure 1B) suggesting that the observed activity is possibly attributable to the reported adjuvant effect of the Der p 2 allergen inseparable from its endogenous LPS cargo [13]. The failure of the HDM extract treated by trypsin to elicit a specific response further supports the contribution of HDM protein components (most probably the Der p 2 allergen), to the TLR4 dependent activation of the TLR4/MD2-CD14 transfected HEK-cells (Figure 1D).

In a similar way, HDM extract stimulation of the TLR2 transfected HEK cells expressing endogenous level of the co-receptors TLR1 and TLR6 induces a clear dose-dependent activation strongly supporting the specificity of the response (Figure 1C). Furthermore, trypsin drastically affected the TLR2-mediated effect of the HDM extract (Figure 1D), indicating again that the TLR2 agonist activity of HDM is most probably mediated by protein material. In an attempt to identify more precisely these components, purified recombinant Der p 1 and Der p 2 were tested individually. Predictably both TLR2 and TLR4 transfected cells were unresponsive to rDer p 1 but also to rDer p 2 more surprisingly. According Trompette et al. [13], the lack of TLR activity of the rDer p 2 was attributed to a sub detectable level of functionally required LPS due to the production/purification procedure (<0.05 EU/mg). However for still unknown reason, we failed to restore the activity of the rDer p 2 with sub active amount of *E. coli* LPS. Then further studies will be necessary to precise the HDM extract components triggering the TLR2 and TLR4 signaling.

### HDM extract elicits nitric oxide and tumour necrosis factor-α production by MH-S cells through TLR2 and TLR4

**Figure 2:**
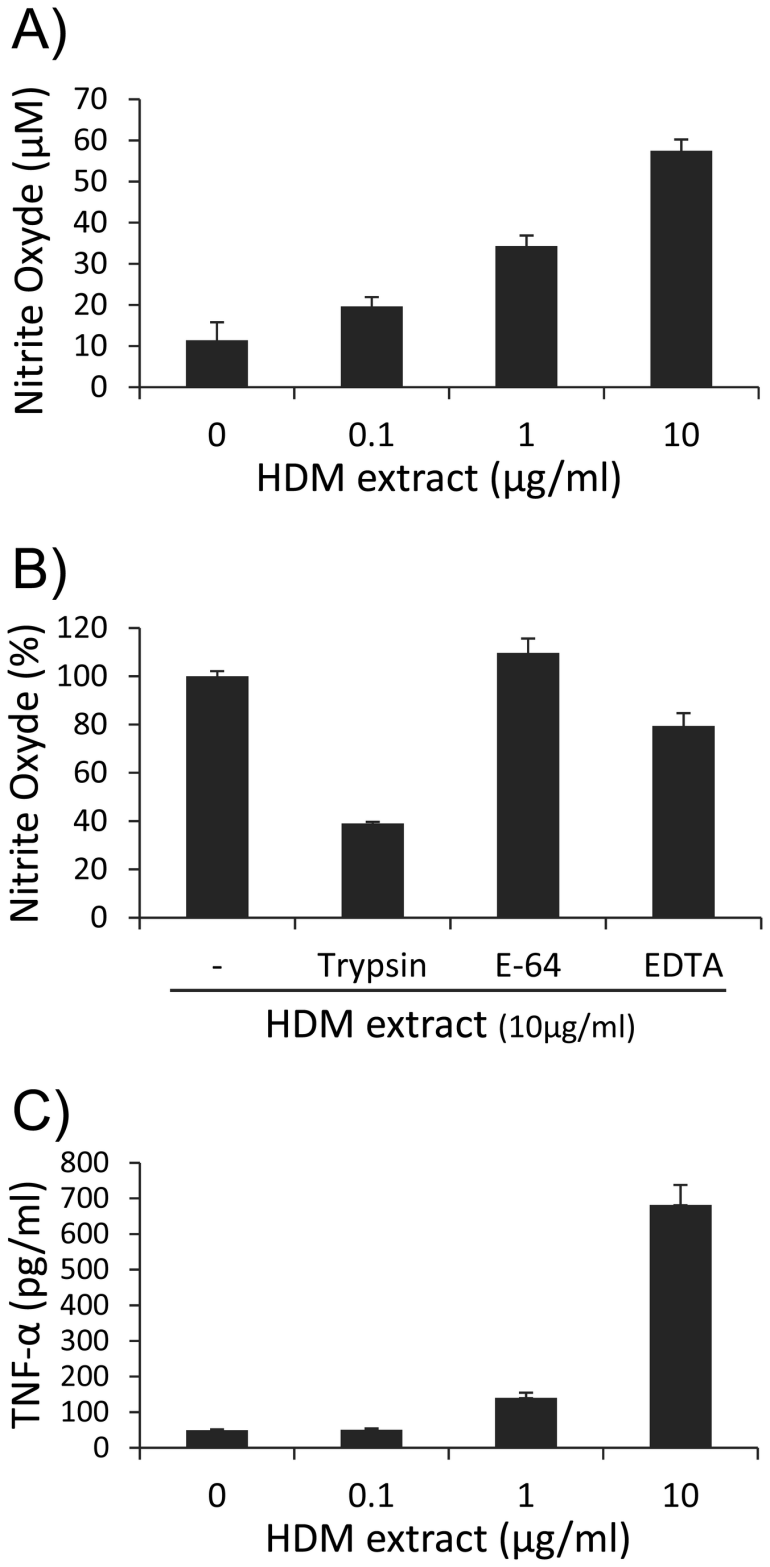
Dose-dependent nitric oxide (NO) production (*A*) and TNF-α production (*C*) by HDM extract-stimulated MH-S cells. MH-S cells were plated at 1x10^6^ cells/mL and challenged with increasing concentrations of HDM extract (0.1, 1 and 10 µg/mL) for 48h. Supernatants were collected and analyzed for NO and TNF-α production. (B) Effect of trypsin-treatment and protease inhibitors on the HDM induced NO production by MH-S cells. HDM extract (10µg/mL) was pre-treated with 5% (w/w) trypsin, 20µM E-64 or 100µM EDTA for 4 hours before addition to MH-S cells for 24 hours. Collected supernatants were assayed for NO production. All samples were analyzed in triplicate. Each bar represents the mean ± SEM from three replicate wells.

A major hallmark of allergic asthma is the local overproduction by alveolar macrophages, of injurious inflammatory response activators, including NO or TNF-α. This damaging effect is specifically activated through the direct recognition of the allergen by the host innate immunity cells and constitutes a natural response independent of any T cell driven allergic susceptibility. Accordingly, treatment of MH-S cells with increasing doses of HDM extract (from 0.1 to 10 µg/mL) triggers a dose-dependent increase of NO production after 24 hours (up to 4 fold higher than the basal NO level of unstimulated cells) (Figure 2A) consistent with the reported overexpression of the inducible NO synthase (iNOs) [17]. Neither polymyxin B, nor inhibitors of the HDM extract associated protease activities affected drastically the NO production. Indeed pre-treatments of the HDM extract by the E-64 inhibitor that specifically blocks the cysteine-protease activity of Der p 1 [17], or by the divalent cation chelating agent EDTA, which is supposed to inhibit the Der p 3, 6 and 9 serine protease activities, altered very moderately (plus 9% and minus 20% respectively) the level of NO production (Figure 2B). In contrast treatment of the HDM extract by trypsin greatly affects its capacity to activate MH-S cells to produce NO (minus 60%).

In a same way, HDM extract strongly enhanced the secretion by MH-S cells of the pleiotropic cytokine TNF-α with a maximum production boost of about 35 fold of the basal production by un-stimulated cells (Figure 2C).

**Figure 3:**
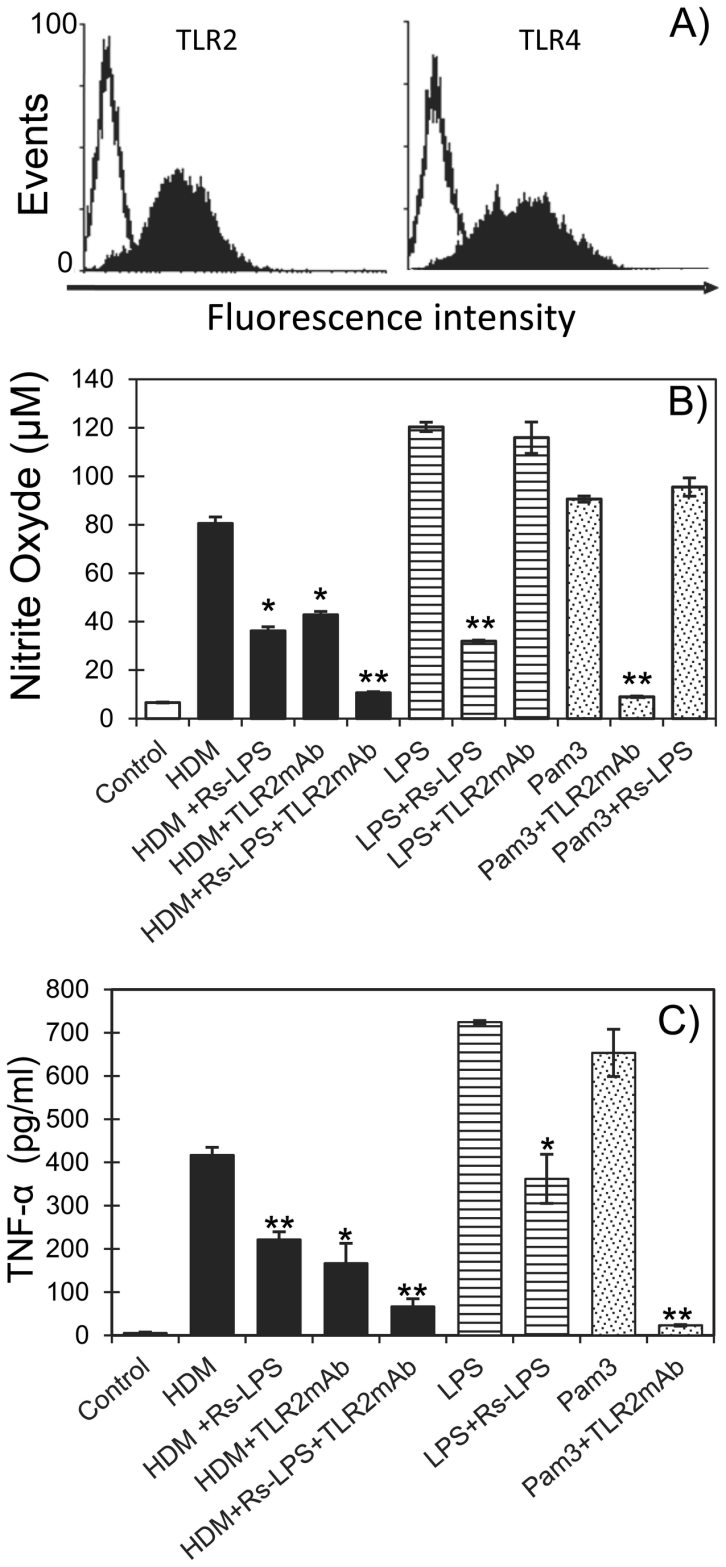
TLR dependent NO and TNF-α production by HDM stimulated MH-S cells. (A) Evidence of constitutive expression of TLR2 and TLR4 in MH-S cells. Cells were incubated with mouse anti-TLR2 and -TLR4 antibodies and analyzed by flow cytometry. Effects of TLR2 and TLR4 antagonists on the HDM extract-induced NO (B) and TNF-α (C) production by MH-S cells. MH-S cells were stimulated by the HDM extract (10 µg/mL), *E. coli* LPS (0.1µg/mL) or Pam3CSK4 (0.01µg/mL) in absence or presence of anti-TLR2 monoclonal antibodies (TLR2mAb) or/and of the TLR4 antagonist LPS from *Rhodobacter sphaeroides* (Rs-LPS, 10µg/mL). After 48h supernatants were collected and assayed for NO and TNF-α production as described in Material and Methods. All samples were analyzed in triplicate and bars represent the mean of the relative values ± SEM from two independent experiments. Statistical analysis was performed using Student’s *t*-test; *p<0.05, **p<0.01.

Then to establish the potential involvement of TLR signaling in the HDM triggered secretion of NO and TNF-α we analyzed the TLR repertoire of the MH-S cells. Indeed, although the presence of TLRs in alveolar macrophage has been largely documented, it is well established that their respective expression may depend on environment physiological factors conditioning the activation state of the immune cell. Flow cytometry analysis of the MH-S cells then revealed that both TLR2 and TLR4 are constitutively expressed (Figure 3A) and thus susceptible to contribute to the specific response of these cells to the HDM extract. The respective mobilization of the TLR2 and TLR4 in the HDM induced NO and TNF-α secretion was assessed by antagonizing selectively each of them separately or concomitantly by specific competitive inhibitors. Interestingly, we found that both NO and TNF-α productions induced by HDM extract are drastically reduced by about 55% (36 *vs* 80 µM) and 47% (222 *vs* 417 pg/mL) by the LPS-TLR4 antagonist ligand from *Rhodobacter spheroides* (Rs-LPS at 10 µg/mL) (Figure 3 B-C). The efficiency and selectivity of the Rs-LPS inhibitory effect is assessed by its capacity to antagonize specifically but incompletely the TLR4 dependent NO and TNF-α production induced by the *E. coli* LPS (minus 73% and 50% respectively) without altering the cytokine production induced by the TLR2 ligand Pam3CSK4. In the same way, anti-TLR2 blocking antibodies reduce severely both the NO and TNF-α productions induced by the HDM extract by about 47% (42 *vs* 80 µM) and 60% (167 *vs* 417 pg/mL) respectively. Again, specificity and efficacy of the blocking effect of these antibodies is confirmed by their capacity to abrogate almost completely the Pam3CSK4 effect (more than 90% and 96% reduction of NO and TNF-α respectively) while they are totally ineffective against *E. coli* LPS.

**Figure 4:**
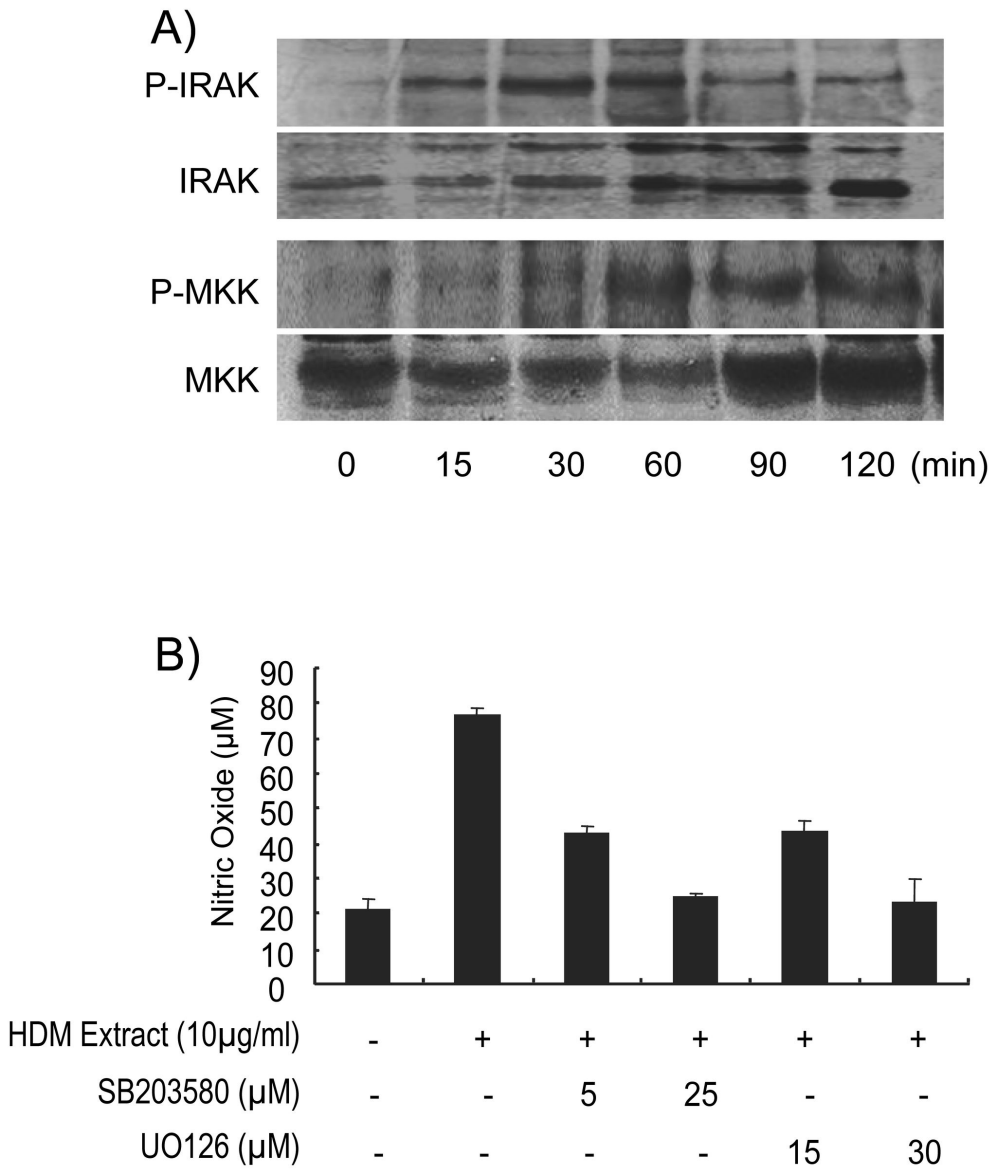
HDM extract-induced NO production involves the TLR2- and TLR4- associated MyD88 signaling pathways and is blocked by p38MAPK and MEK-1, -2 inhibitors. (A) Analysis of phosphorylation of IRAK-1 and MKK. Cellular extracts from HDM extract-stimulated MH-S cells (6x10^6^ cells/mL) were evaluated for p-IRAK-1 and p-MKK protein expression by western blot. (B) Cells were pre-treated with increasing doses of U0126 and SB203580, a MEK-1/2 and a p38MAP kinase inhibitor respectively, for 4 h, and then stimulated with HDM extract for 24 hours. Accumulation of NO in supernatants was evaluated with the Griss Reagent. All samples were analyzed in triplicate. Each bar represents the mean ± SEM of nitrite levels in three replicate wells.

Finally the concurrent use of the two antagonists reveals that the overall amount of NO and TNF-α produced by the MH-S cell likely corresponds to the additive effect of both the TLR2 and TLR4 signaling pathways. Indeed combination of Rs-LPS and anti-TLR2 antibodies lead to a cumulated inhibition of 87% (10.6µM *vs* 80 µM) and 84% (67pg/mL *vs* 417pg/mL) of the NO and TNF-α production respectively. The persistent 15% NO and TNF-α secretion observed in presence of the two antagonists may be attributed to the inability of each of them to reach a total inhibition individually. However it cannot be formally exclude that these residual productions of NO and TNF-α arise from activation through distinct receptors. Engagement of the MH-S TLR2 and TLR4 by the HDM extract has been further verified by assessing the activation of the IL-1R associated kinase (IRAK-1) and MAPK kinase (MKK-3/6) intimately associated to the MyD88-dependent signaling pathway shared by TLR2 and TLR4 (Figure 4A). MH-S cells challenge with HDM extract for increasing periods of time results in a significant phosphorylation of IRAK-1 Thr209 within 15 minutes with a maximum at 60 min evidencing the activation of the MyD88-dependent signaling pathway. Similarly, HDM extract activates MKK-3/6 Ser189/207 phosphorylation but with a slight delay compared to IRAK-1 phosphorylation (Figure 4A). This delay is consistent with the downstream position of MKKs compared to IRAK-1 in the signalisation cascade. Finally, inhibition of the HDM induced NO production by UO126 (selective inhibitor of MEK-1/2) or by SB203580 (selective inhibitor of p38MAPK) clearly demonstrates that the MEK and p38MAPK pathways downstream the IRAK-1-MKK signalling are also mobilized during the TLR2-TLR4 driven response of the MH-S cells to HDM (Figure 4B).

These data obviously evidence that TLR2 and TLR4 both contribute in an additive manner to the production of the inflammatory reaction inducers NO and TNF-α by macrophages exposed to the HDM aeroallergen.

### HDM extract induces a restricted production of the Th1 T-cell activating cytokine IL-12

**Figure 5:**
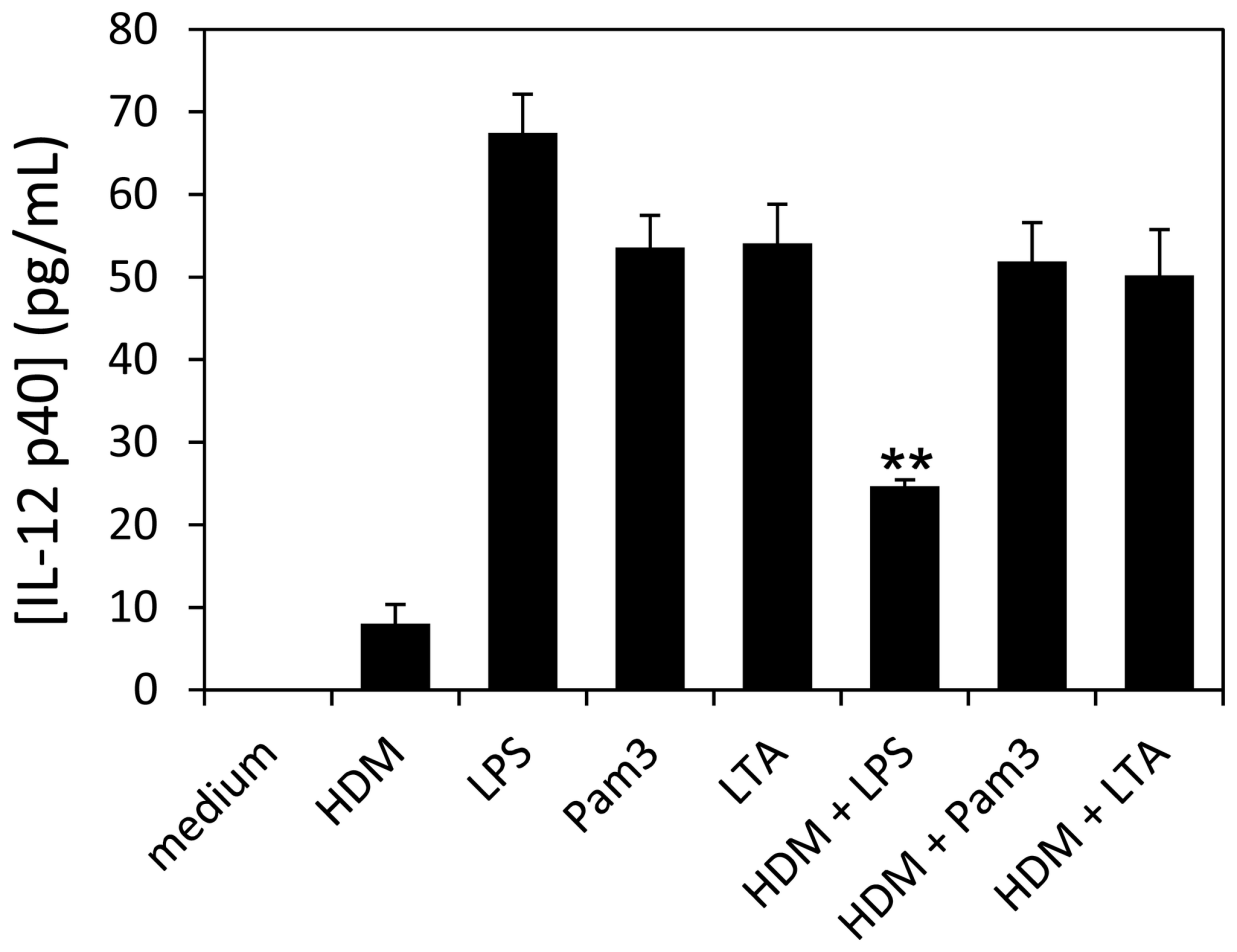
HDM extract induces low production of IL-12p40 and down-modulates the LPS induced of IL-12p40 secretion level in MH-S cells. MH-S cells were incubated with HDM extract or with the individual TLR2 and TLR4 agonists alone (LPS: 0.1µg/mL; Pam3: 0.01µg/mL; LTA: 1µg/mL) or in combination with HDM extract and the IL-12 concentration in supernatants was evaluated at 24h by ELISA after cell centrifugation. All samples were analyzed in triplicate and bars represent the mean ± SEM from duplicate experiments. Statistical analysis was performed using Student’s *t*-test; **p<0.01.

Most of the TLR2 and TLR4 ligands are known to induce also a strong IL-12 production in macrophages and DCs. However, the TLR agonist activity of the HDM extract apparently contrasts with the low level IL-12 secretion reported in allergic asthma patients [18]. Then in order to determine whether the asthma associated IL-12 symptomatic default is due to a T-cell dependent or independent repression mechanism or to a direct failure of the allergenic HDM to trigger macrophage IL-12 secretion, we analyzed the production of IL-12p40 by HDM stimulated MH-S cells. Interestingly, we found that HDM extract (10µg/mL) induces a much reduced IL-12p40 production (8±2pg/mL) by stimulated MH-S cells independently from any T-cell regulation (Figure 5). Moreover we demonstrate that the reduced IL-12 response is specific of the HDM extract since MH-S cells respond positively to TLR activation by TLR2 or TLR4 agonists Pam3CSK4 (0.01µg/mL), LTA (1µg/mL) or LPS (0.1µg/mL), producing high level of IL-12p40: (53.5±4 pg/mL, 54.1±5 pg/mL and 67.4±5 pg/mL respectively, (Figure 5)). We thus verified whether the reduced level of IL-12 could be due to the induction by the allergen of an autocrine repressive production of IL-10 [19]. However no IL-10 secretion could be detected in the culture supernatant of HDM stimulated MH-S cells (minimal limit of detection: 20pg/mL). In a same way, we next thought to verify whether the HDM extract may contains some components able to restrict a TLR-dependent IL-12 secretion through concurrent mechanisms [20] including competing PRR signaling [21]. Unexpectedly, we found that the HDM extract strongly decreased the level of IL-12 produced by LPS- but not by LTA- or PAM3-stimulated MH-S cells. It is noteworthy that a similar inhibition of the LPS-induced IL-12 production (by mycobacterial lipoglycans [21]) has been related to the modulation of the TLR4-dependent activation of the NF-κB transcription family members [22,23].

Thus, we tested whether the HDM extract could affect the nuclear translocation of the NF-κB transcription factors RelA/p65 and c-Rel involved in the activation of the transcription of the immune inflammatory response cytokine gene including IL-12. However immuno-fluorescence microscopy observations and western blot analyses did not reveal any differences in the RelA/p65 or c-Rel nuclear translocation induced by the HDM extract or the individual or combined TLR’s ligands (data not shown). Then the low level of IL-12p40 production of the HDM stimulated cells as well as the inhibition of the LPS-induced IL-12p40 by HDM extract cannot be attributed to any Rel-dependent process.

### TLR2/-4 co stimulatory activities of HDM extract may contribute to down modulate IL-12p40 production

**Figure 6:**
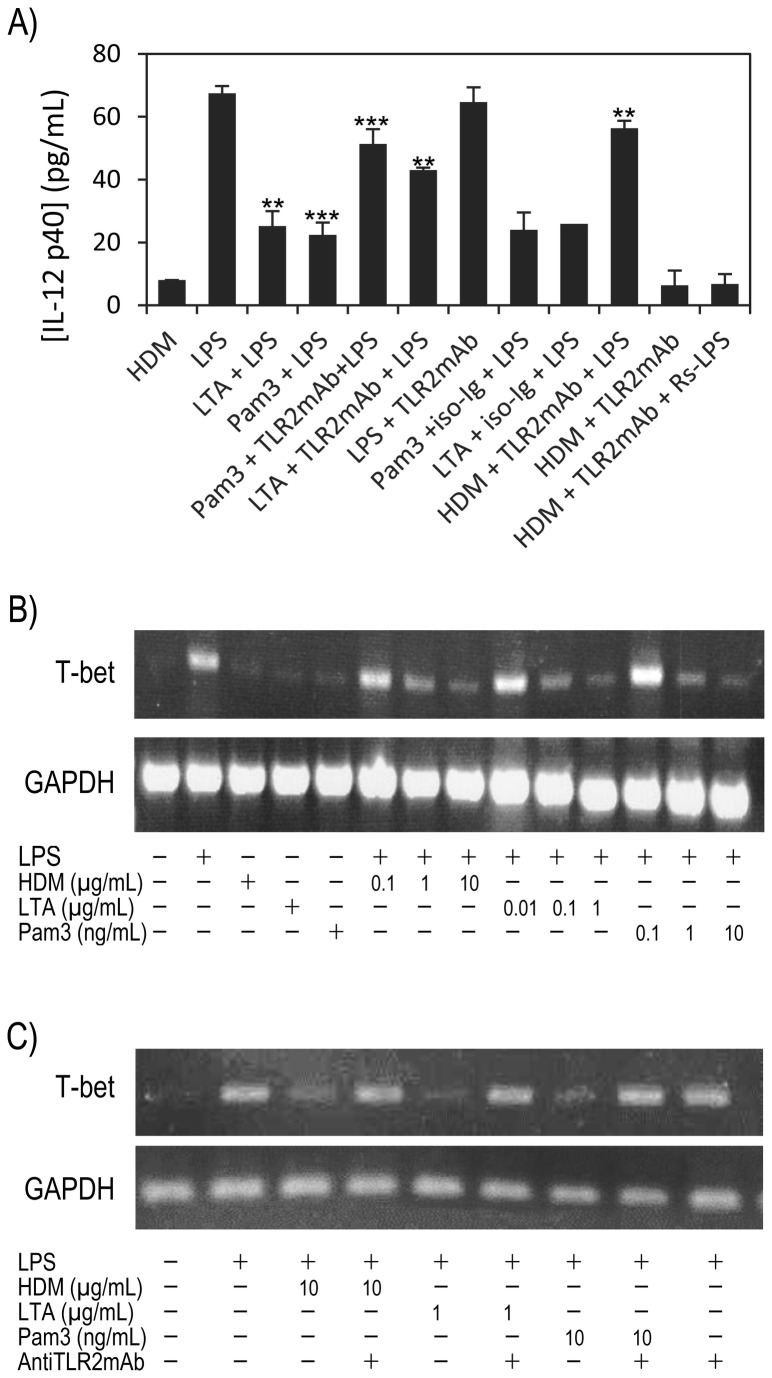
HDM extract prevents LPS-induced expression of Th1-immune response factors in MH-S cells through a TLR2 dependent mechanism. (A) Inhibition of the LPS-stimulated induced MH-S cells IL-12 production by the TLR2 agonists (see legend figure 5 for experimental details; statistical analysis was performed using Student’s *t*-test; ** p<0.01; *** p<0.005). (B) HDM extract inhibition of LPS-induced T-bet expression in MH-S cells. MH-S cells were treated as above except that T-bet expression was evaluated by RT-PCR amplification at 2h and 6h post LPS addition. (C) Suppression by TLR2 antibodies of the inhibitory effect of the TLR2 ligands on the LPS induced T-bet expression (TLR ligands are used at the higher inhibitory concentration tested in B).

Therefore, given that a main difference between HDM extract and the individual TLR agonists resides in the capacity of HDM extract to trigger simultaneously both TLRs, we tentatively hypothesized that this property could be the cause of the failure of HDM extract to induce high level of IL-12p40. To test this assumption MH-S cells were stimulated simultaneously with *E. coli*-LPS (0.1µg/mL) plus LTA (1µg/mL) or Pam3CSK4 (0.01µg/mL). Unexpectedly, we found that concurrent stimulation of the MH-S cells by TLR2 and TLR4 ligands led to a significant reduction (more than 50% decrease) of the amount of IL-12p40 secreted (Figure 6A; LTA + LPS: 25.2±3 pg/mL or Pam3CSK4+LPS: 22.4±1 pg/mL), compared with IL-12 p40 secretion induced by the separate ligands (-53.4% and -58.1% respectively). Then these data obviously reveal that the TLR2 and TLR4 downstream signaling pathways are cross-dependent and that interferences raised by concomitant activation may lead to a down regulation of the IL-12p40 production. This observation is strongly supported by the fact that the IL-12 p40 production of MH-S cells co activated by LPS plus LTA or Pam3CSK4 +LPS can be restored almost completely by anti TLR2 mAb to a level comparable to that observed with LPS alone (Figure 6A). As control, we showed that these anti TLR2 mAb have no effect on the IL-12p40 level induced by LPS (64.6±3 pg/mL) and that unrelated isotype antibodies (iso-Ig) were unable to restore the IL12p40 secretion of (LPS + LTA) or (LPS + Pam3CSK4) activated MH-S cells (Figure 6A; 25.9±0.3 pg/mL, 24±0.4 pg/mL). This result logically led us to emit the hypothesis that the HDM induced repression of the IL12p40 secretion by LPS-stimulated MH-S cells could be attributed the HDM intrinsic TLR2 agonist activity. Interestingly, this assumption was obviously confirmed by the suppression by anti TLR2 mAb of the inhibition by HDM extract of the LPS induced IL12p40 production. These observations clearly evidence that concurrent triggering of the TLR2 and TLR4 signaling pathways down-modulates the TLR dependent IL-12 secretion in MH-S cells. However, although original and attractive, this finding cannot account totally for the failure of HDM extract to induce by itself a significant level of IL-12p40 secretion. Indeed one should expect TLR2 or TLR4 antagonists to abolish the crossed suppressive effect of the intrinsic competing TLR2 and TLR4 agonist activities of the HDM extract and to restore a IL12 secretion level similar to that observed with individual TLR ligands. However neither the anti TLR2 mAb (Figure 6A: 6.3±2 pg/mL), nor the combination (anti TLR2 mAb + Rs-LPS) (6.7±1 pg/mL) could increase the level of IL-12p40 secretion induced by HDM extract to a level close to that obtained with the LTA, Pam3CSK4 or the LPS alone. Then the reduced level of IL-12 induced by HDM extract cannot be definitely attributed to a repressive correlation resulting from the concomitant activation of the concurrent TLR2 and TLR4 pathways. However it is noteworthy that costimulation of MH-S cells with the canonical TLR2 and TLR4 agonists leads to a reduction of the IL-12p40 secretion level similar to that induced by the TLR2 and TLR4 agonistic activities of the HDM extract.

### HDM extract represses the Th1 T-cell lineage transcription factor T-bet

Independently, several recent studies have evidenced that the early differentiation of immature DCs into Th1 polarizing mature DCs is associated with an increased expression of the Th1 T-cell lineage specific T-box transcription factor T-bet (TBX21) [4,24,25]. Since T-bet is a major effector of the Th1 associated gene transcription regulation program, we wanted to verify whether its expression could be affected by HDM extract in MH-S cells supporting the induction of an early AMs phenotypic polarization toward a Th2 adaptive immune response. Consistently with our hypothesis no T-bet mRNA could be detected upon HDM extract triggering while LPS rapidly induced T-bet transcription in less than 2 hours as shown in Figure 6B. Moreover, HDM extract alters LPS-induced expression of T-bet. Indeed, compared to LPS alone, co-stimulation of MH-S cells with LPS (0.1µg/mL) and HDM extract (0.1 to 10µg/mL) results in a lower level of T-bet transcription at 2 hours and which still persists after 6 hours (data not shown). Interestingly in contrast to the TLR4 ligand, Pam3CSK4 or LTA, the respective agonists of the TLR2 heterodimers TLR2/TLR1 and TLR2/TLR6, do not trigger T-bet transcription at concentration at which they induce IL-12 secretion, then resembling to HDM extract in this regard. Therefore these data strongly suggest that the global issue of the response regarding the induction of T-bet expression is largely dominated by the HDM extract TLR2 agonist effect compared to that of the TLR4 agonist. A corollary of this assumption is that the TLR2 ligands must be able to inhibit the expression of T-bet induced by TLR4 agonists. This hypothesis is supported by the dose dependent inhibition of the LPS induced T-bet transcription observed with increasing doses of the TLR2 ligands Pam3CSK4 or LTA. Moreover the involvement of TLR2 in the down-regulation of the LPS induced T-bet expression is convincingly assessed by the inhibition by the anti TLR2 antibodies of the suppressive effect of the TLR2 ligands (Figure 6C).

So far, little is known about the links between T-bet and IL-12, but it has been clearly established that IL-12 triggers T-bet expression in T cells [26]. Accordingly, we hypothesized that T-bet inhibition could be due to the low level of autocrine IL-12 secretion upon HDM extract exposure. However, addition of exogenous IL-12 did not reverse T-bet response (not shown) indicating that HDM extract inhibition of T-bet is independent from that of IL-12. The existence of distinct upstream signaling pathways is further supported by the fact that TLR2 ligands trigger IL-12 secretion but not T-bet transcription. From a mechanistic point of view, to explain the ability of HDM extract to suppress LPS-induced T-bet expression, we hypothesized that HDM extract could down-regulate surface expression of TLR4/MD2-CD14 complex involved in LPS pro-inflammatory signaling. However, flow cytometry experiments did not reveal any decline in the cell surface level of TLR4 and CD14 as already reported in our previous work [10].

## Discussion

Several epidemiological analyses have highlighted positive associations between TLR2, TLR4 and CD14 (TLR4s co-receptor) gene polymorphisms and allergic asthma susceptibility in human [27-29]. These relations are substantiated by studies in animal model showing that TLR2 and TLR4 microbial ligands LPS or LTA modulate experimental allergic asthma [30-32]. However, evidence of molecular and/or physiological direct interaction between allergens and TLRs are still few. In the present work we report that both TLR2 and TLR4 are recruited by a crude extract of the Der p aeroallergen. Indeed, using TLR transfected HEK cell lines we found that the HDM allergen extract activates the NF-κB regulated gene transcription exclusively in TLR2 or TLR4 equipped cells. To date only two HDM components have been identified as potentially contributing to the TLR dependent activation of the innate immune response to Der p [33]: the HDM tightly associated bacterial LPS agonist of TLR4 [13] and the major allergen Der p 2 reported to activate both TLR2 [14] and TLR4 [13] signaling pathways. However, in this study we could not detect any TLR2 or TLR4 activity of purified Der p 2 (probably because of Der p 2 integrity alteration resulting from the purification process [13]) and the HDM components responsible of the TLR dependent responses herein remain to be identified [34].

Interestingly, we demonstrate in a mouse alveolar macrophage cell line model (MH-S), that HDM induced NO and TNF-α productions are dependent of both TLR2 and TLR4 signaling pathways activation and mobilize IRAK-1, MKKs phosphorylation and nuclear translocation of the NF-κB transcription factors Rel-A and c-Rel. However despite the sharing of several signal transduction effectors and pro-inflammatory molecules, HDM was found to trigger an activation pattern of the MH-S cells which differs from that induced by the individual TLR2 and TLR4 canonical ligands mainly by a strongly reduced IL-12p40 secretion level. This failure of the HDM extract to induce IL-12 was attributed in part to its capacity to trigger simultaneously both TLR2 and TLR4 signaling pathways. Indeed we demonstrated that concomitant cell activation by LPS plus LTA or Pam3CSK4 provokes similar significant reduction (>50%) of IL-12 production compared with the activation by the individual agonists. It is worth mentioning that this repressive effect is clearly distinct from the well documented cross tolerance effect occurring under experimental schemes involving successive challenges [35]. Moreover it is to note that the low level of IL-12 induced by HDM highlights the fact that NF-κB activation/nuclear translocation is required but not sufficient to induce IL-12p40 expression while it activates TNF-α and iNOs genes transcription.

Several complex but not fully elucidated mechanisms can account for this apparent discrepancy in the respective regulation of those genes, including complex chromatin structure remodeling of the *il-12p40* promoter [36]. This process occurs independently of the nuclear translocation of the NF-κB c-Rel subunit but depends on the nature of the TLR signaling [37,38]. Thus, further investigations will be necessary to verify whether the oddly low IL-12p40 production induced by the concurrent activation of TLRs 2 and 4 by HDM or by TLR2/-4 ligand combination originates from alterations of the epigenetic processes controlling the accessibility of the *il-12p40* promoter to the transcription regulating factor NF-κB.

In parallel, we show that the HDM extract, as LTA or Pam3CSK4, does not activate the expression of the transcription factor T-bet unlike LPS. This observation strongly suggests that TLR2 agonist moiety of HDM has a dominant repressive effect on the expected T-bet expression resulting from TLR4 triggering. This assumption is supported by the significant reduction of LPS induced T-bet expression provoked by the concurrent triggering of LPS activated cells with HDM or TLR2 agonists. Putative expression and roles of T-bet in macrophages have not been documented, but in keeping with the recent report of its involvement in the regulation of DCs differentiation [4] and in the epigenetic activation of the transcription of the *Ifng* locus associated to Th1 T cell differentiation [39], we hypothesize that the lack of expression of T-bet in allergen-activated lung alveolar macrophages, precludes their differentiation into a phenotype able to activate an effective Th1 response.

In conclusion, our observations not only confirm that recognition and response of the innate immune system to the Der p aeroallergen can mobilize both TLR2 and TLR4 signaling but most importantly we evidence a cross talk between the TLR2 and TLR4 pathways which we propose to contribute to the fine tuning of the macrophage phenotypic polarization through interplays between gene transcription activating factors such as NF-κB, T-bet and AP-1 [10]. Hence, the TLR cell repertoire, and in particular the balance between TLR2 and TLR4 at the cell surface may be crucial for determining the subset of inflammation effectors produced by the innate immune system cells and that will decide for the fate of the adaptive immune response toward a Th1 pro-inflammatory or a Th2 allergic reaction.

Our results highlight the necessity to further investigate the roles of these major innate immune system receptors and the interplays between them or with other specific innate immune cells receptors (such as C-type lectins), in the determinism of the immune response skewing towards a Th1, Th2 or Th17 T cell mediated response. In the light of the promising development of new TLR-targeted immunotherapies of allergic airway diseases [40,41], this might have major incidence on how to develop new pharmaceuticals to exploit these receptors and their associated signaling pathways to treat asthma and other allergic diseases.

## Materials & Methods

### Cell culture

Mouse alveolar macrophage MH-S cells were purchased from the American Type Culture Collection (ATCC, Rockville, MD, USA). Cells were cultured in RPMI 1640 medium supplemented with 10% fetal bovine serum (FBS), 50 units/mL penicillin, and 0.05 mg/mL streptomycin, 20 mM L-Glutamine, 10 mM HEPES, 10 mM sodium pyruvate, 1% (w/v) Glucose and 50 µM 2-ME at 37°C in 5% CO2. Before each experiment cells were washed with PBS and suspended in fresh RPMI medium (Gibco BRL).

### Reagents

Der p (HDM) extract (1 g lyophilized whole body extract in ether; Allergon, Engelholm, Sweden) was dissolved in pyrogenic-free isotonic saline buffer, filtered through a 0.22 µm filter, and stored at 70 °C before use [10]. The LPS concentration of the preparations was <0.96 EU mg^-1^ of HDM extract (Limulus amebocyte lysate test; E-Toxate; Sigma-Aldrich). Baculovirus rDer p 2 was purified as previously described [42] and solubilized in endotoxin-free water at 5µg/mL. LPSs (*E. coli* 055:B5, Sigma-Aldrich; *E. coli* K12, Invivogen; *R. sphaeroides*, Invivogen) and Trypsin (Sigma-Aldrich), Pam2CSK4 (InvivoGen), Pam3CSK4 (InvivoGen), lipoteichoic acid (LTA, InvivoGen), EDTA (Sigma-Aldrich) and E-64 (Sigma-Aldrich) were dissolved in endotoxin-free water. PMSF (Fluka) was dissolved in anhydrous isopropanol and U0126 (Invivogen) and SB203580 (InvivoGen) in DMSO. Reagents were filtered through 0.22-µm filters and stored at -70°C before use.

### Stimulation of MH-S cells and determination of NO production

MH-S cells were plated at 1 x 10^6^ cells/mL in 6-well plates (Falcon) and incubated with increasing concentrations (0.1, 1.0, 10 µg/mL) of HDM extracts 24 h or 48 h. To tests the specificity of the HDM extract effect, this was pre-incubated with 5% Trypsin (w/w according HDM protein content determined by Abs. at 280nm, E-64 (20µM) or EDTA (100µM) for 4 h before addition to the cells for 24 h. The collected supernatants were assayed for NO production as described [30] using Griess reagent (1% (w/v) sulfanilamide, 0.1% (w/v) N-1-naphtylethylenediamine, and 5% (v/v) phosphoric acid).

### ELISA for cytokines

Concentration of cytokines and chemokines in supernatants of stimulated MH-S cells was determined with DuoSet sandwich ELISA assay kits from R & D System using matching monoclonal antibody (mAb) pairs for TNF-α (DY410), IL-12 (DY499) and IL-10 (DY417) with detection limit range from 20 to 2000 pg/mL (according manufacturer).

### Western Blot Analysis

MHS Cell were lysed in lysis buffer (1% Triton X-100, 150 mM NaCl, 10 mM Tris, pH 7.5, 5 mM EDTA 5mM NaN3, 10 mM NaF, 10 mM sodium phosphate) in the presence of PMSF, protease inhibitor mix and the phosphatase inhibitor cocktail I (Sigma-Aldrich). Whole cell extracts were prepared and resolved by SDS-PAGE electrophoresis. Gels were then transferred onto nitrocellulose membranes using a semi-dry cell transfer apparatus (Bio-Rad) and revealed according to the manufacturer’s instructions with anti-IRAK (4362), anti-phospho-IRAK (4361), anti-MKK3/6 (9232), anti-phospho-MKK3/6 (9231) (all from Cell Signaling Technology, Inc.). Membranes were then incubated with horseradish peroxidase-conjugated goat anti-rabbit or anti-mouse antibodies and developed by chemiluminescence (ECL, Amersham; Arlington Heights, IL). All antibodies were used at the working dilutions recommended by the manufacturers.

### Flow cytometry analysis

MH-S cells (1 x 10^6^) were incubated with anti-CD16/CD32 mAb (Fc blocker, 2.4G2, BD PharMingen, San Diego, CA) followed by PE-conjugated anti-TLR4 (AMS-32.1, Santa Cruz Biotechnology, Inc) and anti-TLR2 (T2.5, ebioscence) mAbs for 30 min on ice. After three washes with PBS, cells were stained with FITC-conjugated anti-mouse IgG and suspended in 200 µL PBS for flow cytometry analysis using a FACScan flow cytometer (Becton Dickinson Immunocytometry, San Jose, CA). For each sample, 15,000 events were obtained. Isotype-matched mAb-stained cells were used as a background control in all experiments.

### Toll Like receptor stimulation assay

The HDM extract screening for TLR agonist was performed using the Invivogen Toll like receptor (TLR) transfected human embryonic kidney (HEK 293) cell lines expressing each a different TLR (HEK-Blue^TM^ TLR2, 3, 4, 5, 7, 8, and 9) as well as the secreted alkaline phosphatase gene under the control of a modified human IL-12p40 promoter that can be induced by NF-κB. The amount of secreted alkaline phosphatase, in arbitrary units, was determined using the SEAP Reporter Assay Kit (measurements were performed by the TLR screening service of InvivoGen, Toulouse, France). HEK293 cells were plated in 96-well plates (100 µl total volume) at a concentration of 3-5 × 10^4^ cell/well, then 10 µl of HDM extract were added and incubated at 37°C for 18 h. NF-κB activation was then quantitated spectrophotometrically.

An initial screening was performed (in duplicate) by challenging the 7 HEK-TLR cell lines with 10 µg/mL of HDM extract and measuring the induced alkaline phosphatase activity. Negative controls were mock- induced cells and positive controls cells stimulated with the following ligands: Pam2CysK4 (10 ng/mL) for TLR2, poly I:C (100 ng/mL) for TLR3, LPS-Ek (100 ng/mL) for TLR4, flagellin (100 ng/mL) for TLR5, R848 (10 µg/mL) for TLR7 and -8, and ODN 2006 (10 µg/mL) for TLR9 (InvivoGen). Activation of TLR4 was further assessed in HEK293 cells that were stably transfected with TLR4, myeloid differentiation protein 2 (MD2) and CD14 (HEK293-TLR4/MD2/CD14). The HDM extract dose–response curves for TLR2 and TLR4 activation were completed in triplicate and compared to those obtained with Pam2CSK4 and Ec-LPS.

### Semi-quantitative RT-PCR amplification

RT-PCR amplification was performed to estimate mRNA expression of T-bet, MDC/CCL-22, and TARC/CCL-17. Total cellular RNA was extracted from pooled MH-S samples with the RNeasy Total RNA kit, (Qiagen, Hilden, Germany) and reversed transcribed with the High-capacity cDNA reverse transcription kit (Applied Biosystems). PCR amplification (Applied Biosystems PCR) was performed with 1 µl DNA. Rounds of amplification and annealing temperatures were optimized for each primer pair. PCR products were loaded onto 1.5% agarose gels and stained with ethidium bromide. To control for sample-to-sample variation, the amount of input DNA was first adjusted to obtain comparable levels of GADPH transcription before PCR amplification. Gene-specific primer pairs (sense and antisense) were as follows:

mT-bet, 5’-TGCCTGCAGTGCTTCTAACA-3’ and 5’-TGCCCCGCTTCCTCTCC AA CCAA-3’,

mTARC, 5’-TTGTGTTCGCCTGTAGTGCAT-3’ and 5’-CAGGAAGTTGGTGAGCTGGTA-3’,

mMDC, 5’-GGTCCCTATGGTGCCAATG-3’ and 5’-TTATCAAAACGCCAGGC-3’

mGAPDH, 5’-GTCTTCACCACCATGGAG-3’ and 5’-CCAAAGTT GTC A TGGAT GACC-3’.

### Statistical analysis

Cytokine contents in MH-S supernatants and NO measurements are expressed as means + SEM and the statistical analyses were performed by Student’s *t*-tests: p value < 0.05 was considered significant.
